# Classification of Platelet‐Activating Anti‐Platelet Factor 4 Disorders

**DOI:** 10.1111/ijlh.14486

**Published:** 2025-05-13

**Authors:** Theodore E. Warkentin

**Affiliations:** ^1^ Department of Pathology and Molecular Medicine McMaster University Ontario Canada; ^2^ Department of Medicine McMaster University Ontario Canada

**Keywords:** autoimmune, heparin‐induced thrombocytopenia and thrombosis (HITT), platelet factor 4 (PF4), vaccine‐induced immune thrombocytopenia and thrombosis (VITT), VITT‐like monoclonal gammopathy of thrombotic significance (VITT‐like MGTS)

## Abstract

**Introduction:**

The prototypic anti‐platelet factor 4 (PF4) disorder—heparin‐induced thrombocytopenia and thrombosis (HITT)—features immunoglobulin G (IgG) class antibodies that activate platelets, monocytes, and neutrophils in a mainly heparin‐dependent fashion via Fcγ receptor‐dependent cellular activation. The identification in 2021 of an ultrarare HITT‐mimicking disorder, vaccine‐induced immune thrombocytopenia and thrombosis (VITT)—triggered by two different adenoviral vector vaccines—abruptly broadened the spectrum of recognized anti‐PF4 disorders.

**Objective:**

To classify platelet‐activating anti‐PF4 disorders, both HITT/HITT‐like and VITT/VITT‐like.

**Methods:**

Literature was reviewed from the perspective of a researcher‐clinician involved in identifying novel anti‐PF4 disorders.

**Results:**

Atypical presentations of HITT with proximate heparin triggers but which evince heparin‐independent platelet‐activating properties (“autoimmune HITT”) have been recognized since 2001; heparin‐independent platelet‐activating properties also characterize HITT‐mimicking disorders with undefined non‐heparin triggers (e.g., post‐knee replacement “spontaneous HITT”). Antibodies identical to those of (vaccine‐induced) VITT can rarely be triggered by natural adenovirus infection. HITT and VITT antibodies recognize different epitopes on PF4. All the aforementioned anti‐PF4 disorders are acute, transient, and self‐limited. Recently, however, chronic anti‐PF4 disorders featuring potent VITT‐like properties of monoclonal proteins (M‐proteins) have been identified: this oftentimes treatment‐refractory entity, named “VITT‐like monoclonal gammopathy of thrombotic significance” (VITT‐like MGTS), dramatically expands the clinical spectrum of recognized anti‐PF4 disorders. Anti‐PF4 disorders with heparin‐independent platelet‐activating antibodies, whether HITT or VITT, may require management strategies beyond anticoagulation alone, including high‐dose intravenous immunoglobulin (IVIG) or (for VITT‐like MGTS) the Bruton's tyrosine kinase inhibitor, ibrutinib.

**Conclusion:**

Clinicians and laboratorians require knowledge of the rapidly broadening spectrum of recognized acute and chronic anti‐PF4 disorders.

## 1 Introduction

Since the initial recognition in 1973 of immune heparin‐induced thrombocytopenia (HIT) [1]—also known as heparin‐induced thrombocytopenia and thrombosis (HITT)—and the subsequent discovery in 1992 of platelet factor 4 (PF4)/heparin complexes as the target antigen of HIT [2], any concept of an “anti‐PF4 disorder” would have been synonymous with classic HIT. Here, the key concept is that heparin‐induced platelet‐activating antibodies of immunoglobulin G (IgG) class exert pathological effects in a heparin‐dependent manner [3]. Affected patients develop thrombocytopenia that begins several days following initiation of heparin (reflecting time to antibody formation), with a high proportion of patients (at least 50%) developing thrombosis, either venous or arterial (occasionally both), and sometimes even microvascular thrombosis [3, 4, 5]. In 2001, this concept of HIT as a disorder limited to heparin‐dependent antibodies was expanded, as a highly atypical subset of HIT, initially named as “delayed‐onset HIT,” was described [6], whereby onset of HIT begins several days after stopping heparin, and where the pathogenic antibodies could be shown to activate platelets in a heparin‐*independent* fashion. Subsequently, delayed‐onset HIT was included within a subset of atypical HIT presentations named “autoimmune HIT,” or aHIT (for review: [7]). Just 7 years later (2008), it was posited that a disorder both clinically and serologically identical to HIT—named “spontaneous HIT (SpHIT)”—could be caused by triggers other than heparin or other pharmacological polyanions [8]. All of these “anti‐PF4/heparin disorders” (or, more generically, anti‐PF4/polyanion disorders) are based upon the recognition that heparin and certain other polyanions can create epitopes on PF4 which are recognized by HIT antibodies. A simple pathophysiological model became established: [9] anti‐PF4/polyanion antibodies (irrespective of their trigger) are detectable by simple PF4/polyanion enzyme‐linked immunosorbent assays (ELISAs); moreover, if these antibodies are indeed pathogenic, this can be demonstrated directly by their reactivity profiles in specialized platelet activation assays.

However, in 2021, there emerged the astonishing finding that two adenoviral‐vector vaccines developed to counter the Covid‐19 pandemic could rarely trigger an acute thrombocytopenic and thrombotic disorder [10, 11] that strongly resembled HIT, as follows: (a) an inciting trigger (adenoviral‐vector Covid‐19 vaccine); (b) thrombocytopenia; (c) thrombosis; (d) activation of coagulation (e.g., greatly elevated d‐dimer levels); (e) time delay between vaccination and resulting thrombocytopenia and/or thrombosis (consistent with vaccine‐induced immune‐mediated adverse reaction); and (f) positive testing in PF4/polyanion ELISAs (for review: [12]). This ultrarare complication of vaccination was named “vaccine‐induced thrombocytopenia and thrombosis (VITT)” [11]. As with HIT, VITT antibodies are also detectable by platelet activation assays; however, unlike HIT assays—where heparin is added to enhance antibody detectability—in VITT assays, addition of PF4 enhances VITT antibody detectability, whereas heparin (even in low concentrations) usually inhibits VITT antibody‐induced platelet activation (for review [13]). Subsequently, VITT antibodies were shown to recognize distinct epitopes in comparison with HIT antibodies [14]. Thus, there has now emerged the broad concept of two distinct prothrombotic anti‐PF4 disorders, subdivided into HIT and VITT (and mimicking) entities.

The aim of this brief review is to present a high‐level classification of this rapidly emerging field of platelet‐activating anti‐PF4 disorders. To emphasize the clinical and laboratory similarities between HIT and VITT, I will subsequently refer to HIT by its commonly used abbreviation, “HITT.” Note that the term “HITT” in this article does not necessarily indicate the presence of thrombosis in any individual patient (although in my experience the majority of HITT patients do have associated thrombosis as a consequence of their having developed pathological HITT antibodies).

## 2 Classification of Anti‐PF4 Disorders

Before classifying anti‐PF4 disorders, I will consider the target of the immune response, PF4.

### 2.1 PF4

PF4 is a highly cationic, tetrameric, globular chemokine protein of 32 kDa comprised of two homodimers [15]. A homodimer is comprised of two proteins of identical primary structure; in the case of PF4, a 70‐amino acid subunit with 2 histidine, 3 arginine, and 8 lysine residues (i.e., the 3 cationic amino acids) is key to the binding to PF4 of (anionic) heparin. The dimeric nature of PF4 is likely also crucial to forming large multimolecular complexes with anti‐PF4/heparin and anti‐PF4 antibodies, as a dimeric structure allows two IgG class antibodies to bind to one PF4 molecule. If two or more PF4 molecules are linked with long chains of polyanionic heparin (in the case of HITT pathogenesis), ultralarge PF4/heparin/IgG immune complexes capable of activating platelets via platelet Fcγ receptors are produced. In VITT, highly avid anti‐PF4 antibodies can create multimolecular PF4/IgG without the need for a pharmacological polyanion such as heparin.

**FIGURE 1:**
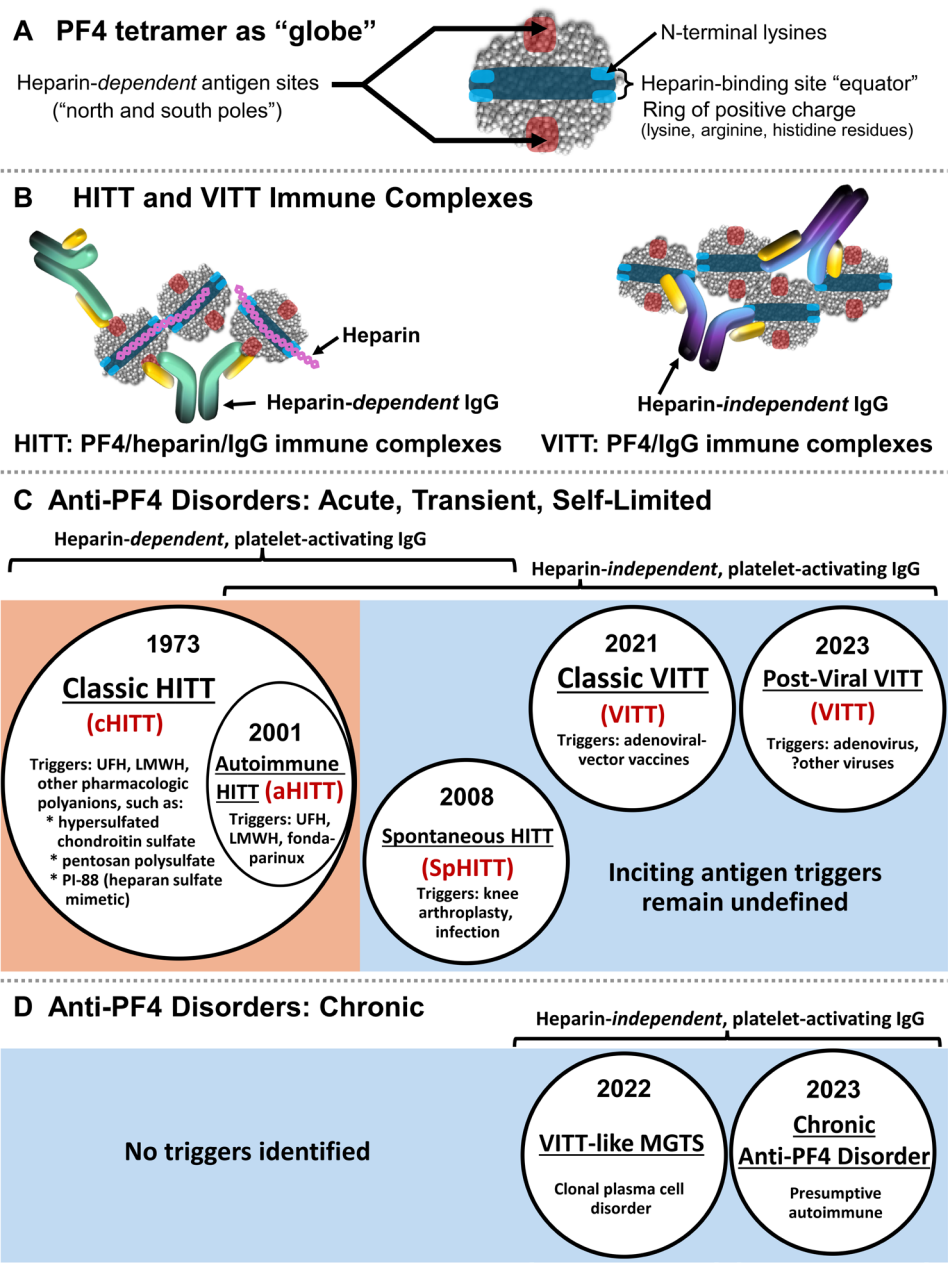
Classification of platelet‐activating anti‐PF4 disorders. (A) PF4 tetramer as “globe.” (B) HITT and VITT immune complexes. (C) Anti‐PF4 disorders: acute, transient, self‐limited. Acute anti‐PF4 disorders include: Classic HITT, autoimmune HITT, spontaneous HITT, classic VITT, and post‐viral (post‐adenovirus) VITT. Note that post‐CMV VITT‐like disorder is included within “post‐viral VITT.” (D) Anti‐PF4 disorders: Chronic. Chronic anti‐PF4 disorders include VITT‐like MGTS and chronic anti‐PF4 disorder (presumptive autoimmune). For each anti‐PF4 disorder, the year of first publication is given.

Figure 1A presents a model of PF4 as a “globe,” and of the distinct regions to which anti‐PF4/polyanion and anti‐PF4 antibodies bind (“poles” and “equator,” respectively) [16].

### 2.2 HITT Versus VITT Antibodies

One obvious definition of HITT antibodies would simply be “antibodies triggered by heparin” whereas VITT antibodies would be defined as “antibodies triggered by an adenoviral‐vector vaccine.” However, it is now recognized that there are triggers other than heparin and vaccines that can lead to anti‐PF4 disorders, raising the question of whether such unusually generated antibodies more closely resemble HITT or VITT antibodies. This raises the conceptual issue of how to distinguish HITT from VITT antibodies. One very complex approach is to identify the epitopes on PF4 recognized by the antibodies: using the globe analogy, heparin‐dependent HITT antibodies recognize polar antigens (north and south poles) whereas VITT antibodies recognize equatorial antigens (crossing points of the equator with the prime meridian and its antimeridian) (Figure 1B).

Another approach is to create distinct PF4/polyanion and PF4 antigens in respective immunoassays. For example, there is a commercial chemiluminescence immunoassay (CLIA) that detects antibodies against PF4/polyanion complexes (PF4/polyanion‐CLIA), with excellent sensitivity for detecting HITT antibodies [17]. A recent modification of this assay—in which PF4 alone (without polyanion) is placed on the plastic beads—can detect the anti‐PF4 antibodies implicated in VITT [18] (however, this novel PF4‐CLIA is not available to the market). Since both HITT and VITT antibodies are recognized by PF4/polyanion ELISAs, but only HITT antibodies are detected by the (commercially‐available) PF4/polyanion‐CLIA, an indirect presumptive suspicion for VITT antibodies is suggested by ELISA‐positive but PF4/polyanion‐CLIA‐negative status.

A more direct approach to recognize VITT antibodies is to use a fluid‐phase ELISA, performed either with PF4 alone or with PF4/heparin complexes [19]. Perhaps by avoiding a wide spectrum of PF4 orientations that are formed on a solid phase (e.g., microtiter plate), the fluid‐phase ELISA (with heparin at concentration of 0.5 U/mL) usually can distinguish between HITT and VITT antibodies [19].

Vayne and coworkers [20] reported another novel approach using the VITT‐mimicking monoclonal antibody, 1E12, to distinguish between HITT and VITT antibodies. In essence, a simple competitive anti‐PF4 ELISA with 1E12 (which inhibits VITT but not HITT antibody binding) could reliably distinguish between these two types of anti‐PF4 antibodies.

### 2.3 Classification of Anti‐PF4 Disorders

Figure 1C,D, list the recognized anti‐PF4 disorders, dividing these into “acute” and “chronic” disorders, and into whether they feature predominantly “HITT/HITT‐like” (mostly heparin‐dependent but sometimes additional heparin‐independent reactivity) or “VITT/VITT‐like” (heparin‐independent) antibodies. Most anti‐PF4 disorders are acute, transient, and self‐limited, that is to say, the antibodies (and ensuing laboratory and clinical abnormalities) develop abruptly, are detectable for only a few weeks or months, and will eventually resolve irrespective of whether the patient is treated or not. However, the existence of chronic anti‐PF4 disorders has become established in the past few years.

### 2.4 Iceberg Model

The “iceberg model” of HITT depicts the concept that while many antibodies are triggered by proximate heparin exposure, only a subgroup with platelet‐activating properties is able to cause HITT itself (“tip of the iceberg”) [21]. The iceberg model can be adapted to illustrate various features of HITT, for example, different frequencies of anti‐PF4/polyanion antibody frequency with different types of heparin given to different patient populations (differing iceberg size) and differing frequency of HITT “breakthrough” for a given heparin‐exposed, antibody‐positive population (differing extent of iceberg protrusion above the waterline) [22]. Per the iceberg model, when evaluating laboratory tests for anti‐PF4/polyanion antibodies in a heparin‐exposed patient, identifying platelet‐activating properties indicates that the patient's thrombocytopenia and/or thrombosis (if present) is likely explained by HITT antibodies. This is a fundamental tenet of both anti‐PF4 disorders, HITT and VITT: acute patient serum will harbor platelet‐activating antibodies.

**FIGURE 2:**
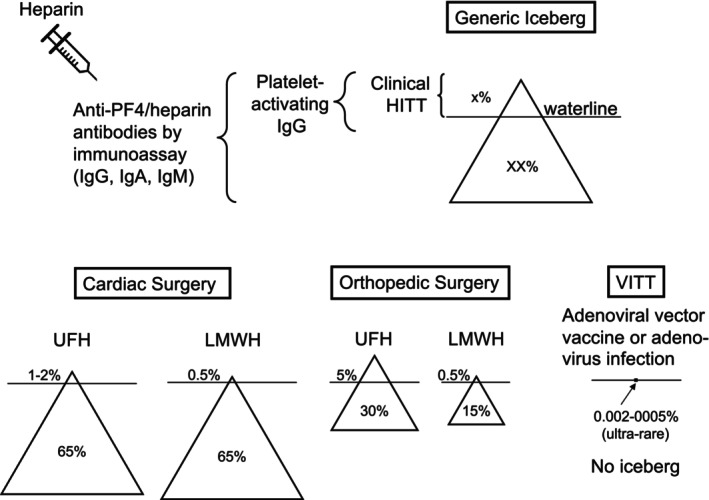
Iceberg model of HITT. In the top part of the figure is shown a generic iceberg. Patients with clinical HITT represent a subgroup of those who form anti‐PF4/heparin antibodies (x% vs. XX%). Below are shown representative icebergs for cardiac surgery and orthopedic surgery, with the former showing a high frequency of anti‐PF4/heparin immunization (~65%), but a relatively low breakthrough of clinical HITT, and with the latter showing a lower frequency of immunization (30% for UFH, 15% for LMWH) but a higher breakthrough corresponding to a higher frequency of clinical HITT (5% and 0.5%, respectively). In contrast, for VITT, there is no apparent iceberg, as the very few patients who develop VITT (0.002%–0.0005% of vaccine/virus‐exposed individuals) do not occur in a background of anti‐PF4 antibodies. Note that the icebergs depicted in this figure do not distinguish between HITT patients who do or do not develop associated thrombotic events. HITT, heparin‐induced thrombocytopenia and thrombosis; IgG, immunoglobulin G; LMWH, low‐molecular‐weight heparin; PF4, platelet factor 4; UFH, unfractionated heparin; VITT, vaccine‐induced immune thrombocytopenia and thrombosis.

Four studies [23, 24, 25, 26] have evaluated the frequency of PF4‐dependent immunoassay positivity events following vaccination with adenoviral vector vaccines. Overall, relatively small percentages of vaccinees had positive PF4‐dependent ELISAs detected before and after vaccination, with relatively few subjects exhibiting “seroconversion”; moreover, in one study [24], antibody frequency was similar between individuals who received ChAdOx1 nCoV‐19 (vaccine implicated in VITT) versus those who received BNT152b2 (mRNA vaccine not implicated in VITT). None of the immunoassay‐positive sera exhibited platelet‐activating properties. Given low levels of anti‐PF4/polyanion antibody positivity known to occur in normal subjects [27], these vaccination studies do not point toward a general anti‐PF4 immune response being triggered by these vaccines. Thus, the “iceberg” phenomenon that is so characteristic of HITT does not appear relevant for VITT pathogenesis. In other words, VITT is caused by a phenomenon whereby through ultrarare mechanisms a patient forms the highly pathogenic VITT antibodies, without any evidence that VITT occurs among a much more common subset of patients who have formed a characteristic immune response triggered by a given vaccine. Figure 2 compares and contrasts HITT and VITT with respect to the iceberg model.

In the remainder of this article, I will provide thumbnail sketches of these various anti‐PF4 disorders. List 1 provides an outline of the classification of anti‐PF4 disorders discussed.

List 1 **Classification of anti‐PF4 disorders.**

ACUTE

HITT/HITT‐like

HITT (UFH, LMWH, fondaparinux)

Classic HITT

Autoimmune HITT

Spontaneous HITT

Postknee replacement surgery

Postinfection

No identified trigger

Non‐heparin pharmacological triggers

VITT/VITT‐like

Classic (postvaccine) VITT

Adenoviral‐vector vaccines

?Other vaccines

Post‐viral VITT

Adenovirus

Post‐viral VITT‐like disorder

Cytomegalovirus (CMV)

?Other viruses

CHRONIC

VITT‐like monoclonal gammopathy of thrombotic significance (VITT‐like MGTS)

Chronic autoimmune anti‐PF4 disorder

## 3 HITT and HITT‐Mimicking Disorders

In this section we will discuss classic and autoimmune HITT (i.e., adverse drug reactions caused by UFH, LMWH, or fondaparinux), HITT‐mimicking complications of non‐heparin polyanionic drugs, and (non‐pharmacologic) SpHITT.

### 3.1 Classic HITT

Classic HITT (or simply, just “HITT”) refers to an adverse drug reaction triggered by heparin (or LMWH) whereby the patient forms platelet‐activating heparin‐dependent antibodies of IgG class that cause approximately 1 week later an unexpected platelet count fall (“thrombocytopenia”) that is often accompanied by one or more new thrombotic events [28, 29]. In some cases, if the patient was receiving heparin to treat thrombosis, HITT can manifest as progression or recurrence of the underlying thrombosis.

The magnitude of thrombocytopenia is often moderate rather than severe. In contrast to drug‐induced immune thrombocytopenia (D‐ITP) secondary to quinine/quinidine, sulfa antibiotics, or vancomycin, which features severe thrombocytopenia (< 20 × 10^9^/L), in HITT, at least 90% of patients have platelet count nadir values > 20 × 10^9^/L (the median platelet count nadir in HITT is approximately 60 × 10^9^/L) [30]. This difference reflects the fundamentally different pathogenesis of D‐ITP (platelet clearance) versus HITT (FcγIIA receptor‐mediated platelet activation) [31].

The frequency of HITT‐associated thrombosis is quite high, at least 50% and as high as approximately 75% [3], especially in postorthopedic surgery patients [4]. Compared to a control group, this increase in thrombosis is substantial, for example, relative risk 12.0 (95% CI, 7.0–20.6) [32]. The most common HITT‐associated thrombotic events are venous, most often lower‐limb deep‐vein thrombosis (DVT), pulmonary embolism, and (central venous catheter‐associated) upper‐limb DVT [3, 28]. Less common (< 5% of patients) but well‐described venous thrombotic events are: splanchnic vein thrombosis (mesenteric or portal), adrenal hemorrhagic infarction (presumptive adrenal vein thrombosis), and cerebral venous sinus thrombosis (CVST) [28]. Necrotizing skin lesions at heparin injection sites [28], and post‐intravenous bolus anaphylactoid reactions [28, 33] are also well‐described HITT‐associated adverse events.

Evaluation of serial blood samples in heparin‐exposed patients after orthopedic surgery has revealed many intriguing features of the anti‐PF4/polyanion immune response [34]. These include (a) a hierarchy of anti‐PF4/polyanion antibody classes, with IgG antibodies more often generated than IgA class antibodies, and IgA class antibodies generated more often than IgM class antibodies; (b) antibodies of all three classes are formed contemporaneously, that is, there is no IgM precedence; (c) only patients who develop IgG class antibodies with platelet‐activating properties are at risk of developing HITT; and (d) higher levels of IgG class antibodies—as quantified by optical density (OD) levels by ELISA—are associated with greater risk of developing HITT [34].

Two intriguing temporal phenomena include (e) “point immunization,” in which antibodies form within a discrete period usually after the first exposure to heparin (e.g., first dose of heparin for postoperative thromboprophylaxis) [28, 34, 35]; as well as (f) seroreversion, that is, antibodies become non‐detectable by platelet activation assay within just a few weeks or months of an acute episode of HITT, followed some months later by non‐detectability by ELISA [35, 36]. Intriguingly, for patients who no longer have platelet‐activating antibodies, heparin reexposure only sometimes restimulates HITT antibodies, and—if it does so—it requires at least 5 days to reach pathogenic levels. The window of time to develop a HITT immune response, and to evince the beginning of the platelet count fall, falls within a characteristic time period, between Days 5–10 (inclusive; day of immunizing heparin exposure = Day 0), irrespective of whether it is the patient's first or multiple heparin exposure [35].

Another well‐known feature of classic HITT is the hierarchy of triggers: UFH results in HITT approximately an order of magnitude (tenfold) more often than does LMWH [37, 38].

### 3.2 Autoimmune HITT (aHITT)

I define “autoimmune HITT”—sometimes referred to as “atypical HITT”—as a subset of HITT that is triggered by proximate exposure to heparin (or another identifiable polyanionic drug) [7]. The key differences vis‐à‐vis classic HITT relate to atypical clinical features (described below) with unusually “strong” HITT antibodies that are capable of activating platelets even in the absence of any heparin added to the test system (e.g., serotonin‐release assay). The topic of aHITT was recently reviewed [39].

One atypical presentation of aHITT is “delayed‐onset HITT” [6]. This is the situation where a heparin‐triggered immune response and associated platelet count fall begins after heparin has been stopped, or which continues to worsen after stopping heparin. Another clinical picture is that of HITT‐associated thrombocytopenia that persists for an unusually lengthy period despite stopping heparin (more than 1 week), called “persisting HITT” or “refractory HITT.” [40] Another clinical picture of aHITT is when the only exposure to heparin was through heparin “flushes” [41]. All patients we have evaluated with heparin “flush” HITT had detectable aHITT antibodies. A fourth aHITT scenario is that of fondaparinux‐associated HITT; in our experience, the majority of these patients have associated aHITT antibodies [42].

Although the aforementioned four entities (delayed‐onset HITT; persisting/refractory HITT; heparin “flush” HITT; fondaparinux‐associated HITT) are well‐established as being associated with aHITT antibodies, there may be other clinical scenarios in which aHITT antibodies are implicated, most notably, very severe HITT with overt DIC [43].

Future research is needed to delineate exactly what characterizes aHITT antibodies. To date, their properties have been evaluated in platelet activation assays, with marked activation at buffer control. Epitope mapping shows that aHITT antibodies bind to both HITT and VITT epitopes [14]. But whether the aHITT phenomenon is caused by one type of antibody or different interacting antibodies remains to be determined.

### 3.3 Spontaneous HITT (SpHITT)

The concept of “spontaneous HITT” is when a patient evinces a clinical picture (generally, thrombocytopenia and/or thrombosis) in association with HITT antibodies that do not represent a response to a pharmacological polyanion, such as heparin, LMWH, fondaparinux, or one of the polyanions discussed later in Section 3.4. In 2008, two papers introduced the concept of SpHITT [8, 44]. One report described three patients who developed thrombocytopenia (two with thrombosis) following infection. The other described a patient who developed severe thrombocytopenia and multiple thromboses (including bilateral adrenal hemorrhagic infarction) following knee replacement surgery (with warfarin rather than heparin thromboprophylaxis).

Several publications have corroborated these findings of a HITT‐mimicking disorder despite the absence of proximate heparin exposure (for review [45]). It appears that approximately half of the reported patients with SpHITT have occurred following knee replacement surgery. This suggests that there may be some type of polyanionic trigger: we have suggested that cellular debris may be generated at the surgical site in relation to tourniquet use for knee replacement surgery, which may result in a “bolus” of debris‐associated polyanion entering the circulation when the tourniquet is released post‐surgery.

Most SpHITT antibodies appear to be aHITT antibodies—with heparin‐independent platelet‐activating properties; this explains how the patient can have thrombocytopenia and thrombosis despite the absence of heparin. However, one case of SpHITT involved a patient who developed abrupt thrombocytopenia when heparin was given for the first time in the patient recipient's life: that patient was shown to have wholly heparin‐dependent antibodies. Another conclusion drawn from testing SpHITT sera in the fluid‐phase ELISA showed these to be distinct from VITT antibodies in most instances [19].

### 3.4 Non‐Heparin Pharmacological Triggers

Several pharmacological polyanions have been implicated in causing a prothrombotic, thrombocytopenic disorder akin to HITT in which the triggering polyanion appears to be both the trigger of the immune response, as well as the polyanion that presumptively contributes to polyanion‐dependent platelet activation. These agents include: hypersulfated chondroitin sulfate [33], pentosan polysulfate [46], and the antiangiogenic agent, PI‐88 [47]. There is insufficient data in the medical literature to indicate whether these can trigger a HITT antibodies.

## 4 VITT

A massive paradigm shift occurred with the recognition of VITT in 2021, as an unanticipated consequence of the enormous effort to develop vaccines to control the global Covid‐19 pandemic. The rapid recognition of VITT reflected both its clinical resemblance to HITT (i.e., thrombocytopenia and thrombosis following a proximate trigger) and the detection of VITT antibodies by standard PF4/polyanion ELISAs used for HITT diagnosis. This last feature has certain paradoxical features, as it is now known that VITT antibodies bind to PF4 alone, and not to the heparin‐dependent antigens created when heparin binds to PF4. This raises the key question: why do PF4/polyanion ELISAs detect VITT antibodies? The answer must relate to some PF4 epitopes recognized by VITT antibodies being formed in the solid‐phase ELISAs (performed on a microtiter plate). In contrast, two commercial immunoassays manufactured by Werfen (Instrumentation Laboratory)—the PF4/polyanion CLIA and the latex‐enhanced immunoturbidimetric immunoassay (LIA)—are both insensitive to VITT antibodies, despite their relatively high sensitivity (at least, 95%) to detect HITT antibodies [13]. This remarkable observation clearly indicates that HITT and VITT antibodies recognize distinct epitopes on PF4.

### 4.1 Classic VITT

The two adenoviral‐vector vaccines implicated in VITT—ChAdOx1‐nCoV‐19 (Oxford/AstraZeneca) and Ad26.COV2.S (Janssen/Johnson & Johnson)—have been permanently discontinued from the marketplace, and thus “VITT” per se should no longer occur in the future. However, as discussed in the next section (Section 4.2), an identical disorder as VITT can be caused by viruses, most notably, adenovirus. Hence, going forward, the term “VITT” will likely be used to denote “virus‐induced immune thrombocytopenia and thrombosis.” In this article, I will use the term “classic VITT” when I refer specifically to VITT triggered by an adenoviral‐vector vaccine, and “post‐viral VITT” to denote explicitly the entity triggered by natural infection by adenovirus.

The reader is referred elsewhere to detailed descriptions of classic VITT [10, 11, 12]. However, Table 1 highlights some of the key clinical and laboratory differences between VITT and HITT. Most notably, compared with HITT, VITT features a higher frequency of thrombosis (> 95 vs. ~50%), a higher frequency of CVST (~50% vs. < 5%), a higher frequency of splanchnic vein thrombosis (~20% vs. < 5%), and a higher frequency of overt hypofibrinogenemia (~30% vs. < 5%). Interestingly, for both HITT and VITT, the first dose of heparin or vaccine (if two or more doses are given) appears to be the immunizing dose.

**TABLE 1:** Comparison between HITT and VITT.

Feature	HITT	VITT
Timing of onset after trigger	At least 5 days	At least 5 days
Frequency	**Common (0.2%–10%)** *	Ultrarare (~1/100,000)**
Risk per immunizing exposure	Point immunization***	First dose (> 95%)***
Thrombocytopenia	Yes (median nadir, ~60 × 10^9^/L)	Yes (median nadir, **~30–50 × 10** ^**9**^ **/L**)
Venous and/or arterial thrombosis	Yes	Yes
Cerebral venous sinus thrombosis (CVST)	< 5%	**~50%**
Splanchnic vein thrombosis	< 5%	**~20%**
Adrenal vein hemorrhage (presumptive adrenal vein thrombosis)	< 5%	< 5%
Deep‐vein thrombosis	**~50%**	~20%
Pulmonary artery thrombosis or embolism	~25%	~25%
Limb artery thrombosis	~10%–15%	~10%–15%
Arterial stroke	~5%–10%	~5%–10%
Myocardial infarction	~5%	~5%
Multi‐site thrombosis	~10%–20%	**~30%**
d‐dimer > 4000 FEU	Yes	Yes
Overt hypofibrinogenemia (< 1 g/L)	~5%	**~25%**

*Note:* Respective data suggesting a greater frequency or severity for one anti‐PF4 disorder (HITT or VITT) versus the other anti‐PF4 disorder are indicated in bold text. Data for this table are estimates by the Author based upon relevant literature [3, 4, 5, 11, 28].

* Frequency of HITT highly variable, depending on the type of heparin (unfractionated > low‐molecular‐weight), duration of heparin exposure (> 10 days vs.> 5 days), clinical context (surgical > medical > obstetrical/pediatric), dose of heparin, and so forth.

** Frequency of VITT is ultrarare, but appears to be more common for ChAdOx1 nCoV‐19 versus Ad26.COV2.S.

*** “Point immunization” refers to the first dose of heparin (e.g., in a series of postoperative subcutaneous heparin injections) being the likely trigger of the anti‐PF4/polyanion immune response; for comparison, for the two‐dose regimen of ChAdOx1 nCoV‐19, with each dose given 3 weeks apart, virtually all reported subsequent VITT cases occurred after the first, rather than the second, vaccine dose administered.

As in HITT [35, 36], pathogenic antibody transience (“seroreversion”) is also a well‐documented feature of VITT [48, 49]. However, antibody persistence appears to be somewhat longer in VITT than in HITT [35, 48]. Indeed, there is anecdotal evidence that even after platelet count recovery, persistence of detectable serum platelet‐activating properties beyond a year can occur in some patients who suffered from acute VITT following vaccination [50].

There are reports of vaccines other than Covid‐19 vaccines causing VITT. One is the human papillomavirus (HPV) vaccine (Gardasil) [51]. Sporadic cases of VITT following mRNA vaccines are sufficiently uncommon that they could represent the background rate of anti‐PF4 disorder (akin to SpHITT) rather than representing a causal relationship [52].

An intriguing report by Wang et al. [53] showed that VITT antibodies exhibit stereotypic light and heavy chain third complementarity‐determining region (LCDR3 and HCDR3) amino acid sequences with near perfect light‐chain stereotypy. The authors proposed that these findings point to highly convergent pathways of anti‐PF4 generation following the presumed discrete adenovirus trigger.

### 4.2 Post‐Viral VITT

In August 2023, my colleagues and I reported two patients who developed thrombocytopenia and thrombosis and platelet‐activating anti‐PF4 antibodies with a VITT‐like reactivity profile following proven acute adenovirus infection [54]. Our findings were quickly followed by similar observations [55]. More recently, evidence of mini‐epidemics of post‐viral VITT has been presented [56, 57]. Interestingly, when we collaborated with Wang and colleagues to characterize the anti‐PF4 antibodies purified from post‐viral VITT subjects, it was shown that post‐viral VITT antibodies are essentially identical to those triggered by adenoviral‐vector vaccines [58]. The unique clonotype identified was specified by an identical immunoglobulin lambda variable 3–21*02 (IGLV3‐21*02) light chain paired with a single heavy chain expressing a shared “ED” amino acid motif in the heavy‐chain third complementarity‐determining region (HCDR3). This characteristic antibody clonotype suggests that the VITT immune response follows an aberrant trajectory that begins as a stereotypical immune response presumably triggered by a viral constituent.

Interestingly, when we performed standard serum protein electrophoresis/immunofixation electrophoresis (SPEP/IFE) to investigate for grossly detectable monoclonal paraproteins, these yielded negative results for our post‐adenovirus VITT sera, indicating that antibody levels were most likely less than 0.2 to 0.4 g/L of IgG (i.e., the lower level of detectability of a paraprotein by SPEP/IFE) [59].

The identification of post‐viral VITT is of enormous significance to clinicians, as it means that the lessons learned during the classic VITT mini‐epidemic that occurred during the use of the adenoviral‐vector vaccines remain valid even today: namely that acute thrombocytopenia and thrombosis (especially following suspected or proven viral infection) may be investigated and treated as a potential anti‐PF4 disorder, with several diagnostic and therapeutic implications (see Section 6).

A recent report of a patient with post‐cytomegalovirus (CMV) VITT‐like disorder is intriguing [60]. The patient had a severe and ultimately fatal clinical course including deterioration during anticoagulation. The patient's platelet‐activating antibodies were shown to have both HITT‐like and VITT‐like features. Further, when the anti‐PF4 antibodies were analyzed, they were monoclonal, and found to have a clonotype distinct from that implicated in post‐vaccine/post‐viral VITT. Moreover, the quantity of the anti‐PF4 antibodies in the patient's blood was sufficiently high to be detectable by standard SPEP/IFE. This well‐documented case of post‐CMV VITT‐like prothrombotic disorder clearly indicates that there exists a diverse spectrum of VITT/VITT‐like immune responses that rarely can be triggered by viruses.

## 5 Chronic VITT‐Like Anti‐PF4 Disorders

As mentioned earlier, HITT and VITT are acute, transient, and self‐limited disorders. Thus, it is another paradigm shift to realize that there are chronic VITT‐like disorders recognized in the recent past. It is anticipated that with greater clinical awareness of these entities, many more affected patients will be identified in the future.

### 5.1 VITT‐Like Monoclonal Gammopathy of Thrombotic Significance

In 2021, Andreas Greinacher and I, along with collaborators, reported an unusual patient in Germany who had a chronic disorder characterized by thrombocytopenia and recurrent thrombosis that was refractory to anticoagulation [61]. We reported that the patient had a monoclonal gammopathy detectable by SPEP/IFE that bore VITT‐like anti‐PF4 properties. Subsequently, additional such patients have been reported, and the term “VITT‐like monoclonal gammopathy of thrombotic significance” (VITT‐like MGTS) has been applied to this discrete entity [62, 63]. It is now well established that the M‐protein itself bears VITT‐like platelet‐activating characteristics [64].

**FIGURE 3:**
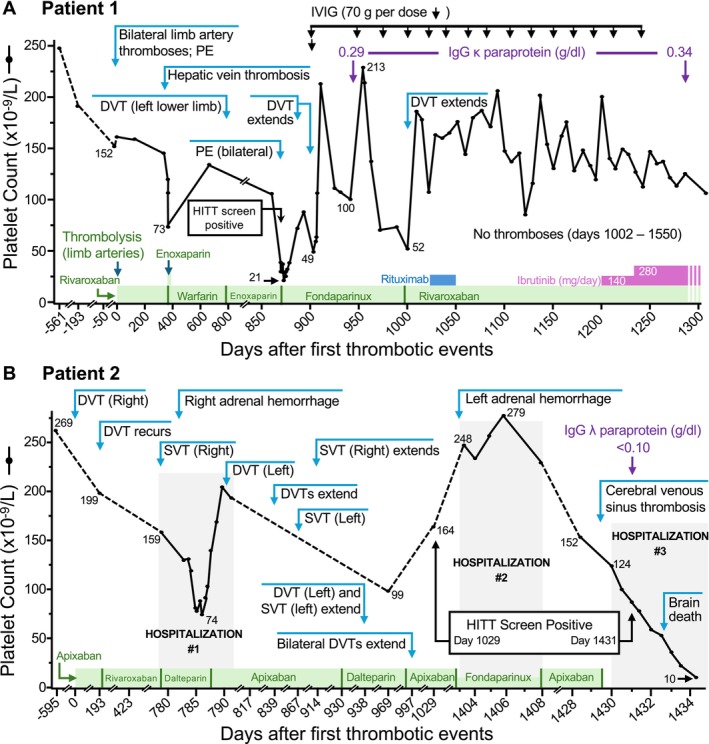
VITT‐like monoclonal gammopathy of thrombotic significance (VITT‐like MGTS): Representative patients. (A) Patient 1 was 60 years old when he presented with bilateral lower‐limb artery thrombosis and pulmonary embolism (PE; Day 0). Over the next 3 years, he developed several recurrent thrombotic events despite therapeutic‐dose anticoagulation, including hepatic‐vein thrombosis, lower‐limb deep‐vein thrombosis (DVT), and bilateral PE, often accompanied by transient declines in the platelet count. High‐dose intravenous immunoglobulin (IVIG) resulted in a transient platelet count increase. Rituximab was ineffective in ameliorating his prothrombotic state. Ultimately, treatment with IVIG was switched to the Bruton's tyrosine kinase inhibitor, ibrutinib, which resulted in an immediate and persisting correction of elevated d‐dimer levels (data not shown), and no further thrombotic events (last follow‐up, 12 months after initiation of ibrutinib). The patient was shown to have an IgG κ paraprotein bearing VITT‐like properties, resulting in a diagnosis of VITT‐like MGTS (data not shown). (B) Patient 2 was 46 years old when he presented with unprovoked right‐lower‐limb DVT. Over the next 4 years, he developed multiple recurrent thrombotic events despite therapeutic‐dose anticoagulation, including DVTs and superficial vein thrombosis (SVT) and right adrenal hemorrhage (presumptive indicator of adrenal‐vein thrombosis) followed 1 year later by left adrenal hemorrhage (with biochemical evidence of adrenal failure), often accompanied by transient declines in the platelet count. Ultimately, he developed fatal cerebral venous sinus thrombosis (CVST), accompanied by a substantial platelet count decline. A blood sample (labeled “Day 1431”) investigated following death revealed an IgG λ paraprotein bearing VITT‐like properties, resulting in a diagnosis of VITT‐like MGTS (data not shown). DVT, deep‐vein thrombosis; HITT, heparin‐induced thrombocytopenia and thrombosis; IVIG, intravenous immunoglobulin; PE, pulmonary embolism; SVT, superficial vein thrombosis (greater saphenous vein). Reprinted, with modifications, with permission from: Wang et al. [64]. Copyright: Massachusetts Medical Society.

There are several intriguing features of these patients. First, the thrombocytopenia may be chronic/progressive or may be intermittent (with exacerbations in platelet count decline often occurring in association with thrombosis) [64]. Second, a wide spectrum of thrombotic events can be seen (see Figure 3A,B) [64]. Third, subsequent thrombotic events can occur even while the patient is receiving therapeutic‐dose anticoagulation with good compliance [64]. Fourth, the patient's serum/plasma contain antibodies with VITT‐like rather than HITT‐like properties. Fifth, the M‐protein levels—which reflect the presence of an abnormal plasma cell clone—can be relatively low, at levels more in keeping with “benign” monoclonal gammopathy rather than multiple myeloma; indeed, one patient with a barely detectable M‐protein (< 1 g/L) died of CVST (the platelet‐activating M‐protein was detected following the patient's demise). Sixth, these patients require treatment beyond anticoagulation alone [62, 63, 64], such as (a) ancillary treatments to ameliorate antibody‐induced platelet activation (e.g., high‐dose intravenous immunoglobulin [IVIG]; tyrosine kinase inhibitor, ibrutinib) or (b) plasma cell‐directed treatment. Seventh, the age of onset of thromboses in several of the patients occurred at relatively young ages (30s through 50s), much younger than one generally expects for clonal plasma cell disorders.

The identification of a chronic prothrombotic hypercoagulability state mediated by VITT‐like anti‐PF4 antibodies has profound implications for the standard work‐up of an underlying prothrombotic disorder (see List 2). Further, one might predict that there will be future patients identified who have VITT‐like MGTS even though a paraprotein will not be detected by standard SPEP/IFE techniques: this is because the lower limit of paraprotein detectability (~0.20 g/L) [59] is considerably greater than the concentration of manufactured VITT‐like monoclonal antibodies that can cause platelet activation under test conditions (15–60 μg/mL [0.015–0.060 g/L]) [65]. In such a (theoretical) patient, despite an M‐protein not being readily identifiable (by SPEP/IFE), the patient will test positive by PF4/polyanion ELISA and have a positive PF4‐enhanced washed platelet activation assay.

List 2 **Proposed hypercoagulability workup (addition of testing for VITT‐like MGTS^a^).**

Screening investigations for disseminated intravascular coagulation (DIC)

Complete blood count, blood film review; prothrombin time, partial thromboplastin time, fibrinogen, d‐dimer (or other fibrin‐specific marker)

Congenital disorders.

Factor V Leiden

Prothrombin gene mutation

Antithrombin activity

Protein C

Protein S

Acquired disorders

Antiphospholipid syndrome (antigen assays; lupus anticoagulant assays)

Paroxysmal nocturnal hemoglobinuria

Jak2‐V617F mutation

Novel VITT‐like monoclonal gammopathy of thrombotic significance (VITT‐like MGTS)^a^

PF4/polyanion ELISA

Rapid HITT assay (e.g., LIA, CLIA)^b^

Platelet activation assays (e.g., serotonin‐release assay [SRA], PF4‐enhanced SRA)

Serum protein electrophoresis/immunofixation electrophoresis (SPEP/IFE)^c^

^a^The indications for including screening for VITT‐like MGTS as part of a hypercoagulability work‐up remain unresolved; the author recommends including screening for VITT‐like MGTS particularly in situations in which atypical or anticoagulant‐refractory thrombosis occurs, intermittent or chronic thrombocytopenia is present (especially if thrombocytopenia is associated with thrombosis), or if thrombocytopenia or thrombosis occurs in a patient known to have a monoclonal gammopathy.

^b^VITT‐like MGTS antibodies typically test negative by LIA and CLIA; accordingly, ELISA‐positive but LIA/CLIA‐negative reactivity may indicate indirectly the presence of VITT/VITT‐like antibodies.

^c^In theory, a patient could test negative for M‐protein by SPEP/IEP, but still have VITT‐like MGTS, based on the threshold for M‐protein detectability by SPEP/IEP (~0.2 g/L) being higher than the minimum concentration of VITT‐like monoclonal antibodies (0.015 to 0.060 g/L) known to cause in vitro platelet activation.

Abbreviations: CLIA, chemiluminescence‐immunoassay; ELISA, enzyme‐linked immunosorbent assay; LIA, latex‐enhanced immunoturbidimetric immunoassay; PF4, platelet factor 4; VITT, vaccine‐induced immune thrombocytopenia and thrombosis.

#### 5.1.1 Neonatal Thrombocytopenia and CVST in Maternal VITT‐Like MGTS

Recently, Häusler et al. [66] reported a neonate with thrombocytopenia (platelet count, 32 × 10^9^/L), CVST, hypofibrinogenemia, and elevated d‐dimer levels who was born to a 35‐year‐old mother with an IgGκ monoclonal gammopathy. The investigators showed that transplacental passage of maternal M‐protein with VITT‐like platelet‐activating properties explained this remarkable clinical picture.

### 5.2 Chronic VITT‐Like Autoimmune Anti‐PF4 Disorder

There is one reported case of a patient with a chronic prothrombotic disorder in which anti‐PF4 antibodies with heparin‐independent platelet‐activating properties were identified [67]. This patient was tested on numerous occasions using standard investigations for M‐protein, and none was detected. This is a presumptive autoimmune anti‐PF4 prothrombotic disorder, in which the prothrombotic state appears to have been ameliorated by giving ibrutinib.

## 6 Treatment Considerations

Detailed discussion of treatment for anti‐PF4 disorders is beyond the scope of this article. However, the existence of numerous anti‐PF4 disorders with heparin‐independent platelet activation properties indicates that treatment approaches besides heparin cessation itself (if heparin is even being given) require consideration.

### 6.1 Ancillary Treatment Besides Anticoagulation

It is known that high‐dose IVIG interferes, through direct competition, with the ability of PF4‐ and IgG‐containing immune complexes to interact with platelet Fcγ receptors, and there is considerable evidence that these can help in “cooling off” the intense prothrombotic state of entities such as aHITT, SpHITT, and VITT. Bruton's tyrosine kinase inhibitory agents such as ibrutinib can decrease signal transduction of platelet activation pathways triggered through Fcγ receptor‐mediated activation and have been shown to ameliorate platelet activation in chronic anti‐PF4 disorders [63, 64]. Finally, for the rare patients in whom M‐proteins are believed to be responsible for clinical disease (VITT‐like MGTS), plasma cell‐directed therapy can eradicate the prothrombotic state if it is successful in eliminating or reducing the plasma cell clone [63, 64].

### 6.2 Anticoagulation: Heparin Versus Non‐Heparin Anticoagulation

A controversial topic is what the ideal anticoagulant ought to be to treat HITT/HITT‐like and VITT/VITT‐like disorders. In general, heparin is proscribed (contraindicated) in patients believed to have HITT, and indeed, heparin administration to a patient with HITT antibodies can have catastrophic consequences (e.g., acute anaphylactoid reactions particularly with intravenous heparin bolus administered to a patient with HITT antibodies) [33]; on the other hand, the beneficial (anticoagulant) effects of heparin can mitigate any potential adverse consequences, especially in heparin‐independent scenarios (e.g., in the author's experience, HITT‐associated warfarin‐induced venous limb ischemia/gangrene typically occurs only *after* heparin has been stopped). Especially given heparin's ability to inhibit VITT antibody‐induced platelet activation at low heparin concentrations, this suggests that heparin (UFH, LMWH) may be effective in patients with VITT. A review of CVST complicating VITT did not find any adverse signal with heparin (vs. non‐heparin) anticoagulation, while finding that high‐dose IVIG was associated with improved outcomes [68]. And of course any heparin “failures” in patients with severe HITT or VITT may not reflect any true adverse consequence of the heparin itself versus the general failure of anticoagulation in a patient with a severe underlying acute antibody‐induced hypercoagulability state.

## Author Contributions

This review article was solely written by Theodore E. Warkentin.

## Ethics Statement

Ethical approval was not sought for the present study because our institution does not require an ethics approval for a review of literature.

## Consent

The author has nothing to report.

## Conflicts of Interest

Theodore (Ted) E. Warkentin, MD, has provided consulting services to Paradigm Pharmaceuticals, Werfen (Instrumentation Laboratory), and Veralox Therapeutics; has received research funding from Werfen (Instrumentation Laboratory); and has provided expert witness testimony relating to heparin‐induced thrombocytopenia and thrombosis (HITT) and non‐HITT thrombocytopenic and coagulopathic disorders.

## Data Availability

Data sharing is not applicable to this article as no new data were created or analyzed in this study.
